# Histology: Normal and Morbid

**Published:** 1899-04

**Authors:** 


HISTOLOGY: NORMAL AND MORBID. By EDWARD K. DUNHAM, Ph. B , M. D., Professor of General Pathology, Bacteriology and Hygiene, in the University and Bellevue Hospital Medical College, New York. Illustrated with 363 engravings. Pp. 448. New York and Philadelphia: Lea Brothers & Co. 1898.

The writer has devoted most of the space in this work to a description of the normal structures and has contented himself with only a brief account of the histology of the more prevalent morbid processes. For the sake of clearness, however, examples of morbid structures have been selected from various parts of the body to illustrate the different phases of the processes that were being outlined. The author divides his work into: Part I., devoted to normal histology; Part II., devoted to histology of the morbid processes and Part III., to histological technique. We know of no text-book on histology that covers the ground so thoroughly and yet concisely and predict for it a successful future. The author is a careful, painstaking worker and he stamps his individuality on every page of the book. Printing and illustrations are of excellent quality.

